# *Bifidobacterium animalis* Subsp. *lactis* PB200 Improves Intestinal Barrier Function and Flora Disturbance in Mice with Antibiotic-Induced Intestinal Injury

**DOI:** 10.3390/nu17101610

**Published:** 2025-05-08

**Authors:** Ganen Wang, Han Gong, Yang Zou, Haijiao Zhang, Xueying Mao

**Affiliations:** 1 Key Laboratory of Functional Dairy, Ministry of Education, College of Food Science and Nutritional Engineering, China Agricultural University, Beijing 100083, China; 2 Tianjin Haihe Dairy Co., Ltd., Tianjin 300300, China

**Keywords:** probiotic, antibiotic-induced gut dysbiosis, intestinal barrier damage, inflammation

## Abstract

**Background/Objectives**: Overuse or misuse of antibiotics could cause adverse effects such as gut microbiota dysbiosis and intestinal barrier dysfunction. Probiotic intervention could effectively alleviate these symptoms. However, the precise efficacy of *Bifidobacterium animalis* subsp. *lactis* PB200 (*B. lactis* PB200) in mitigating antibiotic-induced intestinal injury remains unclear. **Objective:** The aim of this study was to systematically evaluate the effects of *B. lactis* PB200 on intestinal barrier injury and gut microbiota dysbiosis in a murine model of antibiotic-induced intestinal injury. **Methods:** BALB/c mice were administered ceftriaxone sodium via oral gavage for seven consecutive days, followed by probiotic intervention daily via gastric gavage for 4 weeks. **Results:** The results indicated that *B. lactis* PB200 played a positive role in enhancing intestinal barrier function, as evidenced by the restored intestinal morphology, and elevated the expression of tight junctions including ZO-1, Claudin-4 and Occludin (2.76-fold, 4.39-fold, and 2.61-fold, respectively) compared to that in the Model group. *B. lactis* PB200 normalized the levels of serum pro- and anti-inflammatory factors, including IL-1β, IL-6, TNF-α and IL-10, and elevated the diversity and richness of gut microbiota. *B. lactis* PB200 significantly elevated the levels of propionic acid and butyric acid, with increases of 1.67-fold and 2.82-fold, respectively, compared to the Model group. Notably, *B. lactis* PB200 reduced the abundance of *Enterocloster* and increased the abundance of *Parabacteroides*, promoting the rebalance of gut microbiota. **Conclusions:** Taken together, these findings highlighted the significant potential of *B. lactis* PB200 in alleviating intestinal barrier damage and restoring the balance of gut microbiota caused by an antibiotic.

## 1. Introduction

Antibiotics are commonly used in clinical treatment of illnesses caused by pathogenic bacteria. However, they could also deplete commensal microbes and disrupt gut microecology, which lead to adverse effects on gut homeostasis [1]. Ceftriaxone sodium, a β-lactam antibiotic, has been shown to induce gut microbiota dysbiosis following both short-term and long-term administration [2]. Such disruptions resulted in overgrowth of opportunistic pathogens, intestinal barrier damage, and immune dysfunction [3,4]. These consequences significantly impair overall intestinal health. Therefore, it is necessary to understand the mechanisms by which antibiotics compromise intestinal barrier integrity to facilitate the development of safe and effective nutritional interventions.

Maintaining intestinal homeostasis is critical for overall health, and the intestinal barrier plays an indispensable role in this process. Age, diet, and the use of antibiotics could all have an impact on the gut microbiota, which is in a state of dynamic balance. The gut microbiota is essential for maintaining intestinal homeostasis. By producing bioactive metabolites such as short-chain fatty acids (SCFAs), commensal microorganisms competitively inhibit pathogenic colonization while reinforcing intestinal barrier integrity [5]. Furthermore, butyrate enhanced the intestinal epithelial barrier by upregulating expression of Claudin-1 and reversed the decreased expression of ZO-1 and Occludin [6,7]. However, antibiotic-treated mice exhibited a marked reduction in the expression of tight junctions (TJs) [8]. Broad-spectrum antibiotics could affect the abundance of the gut microbiota, leading to adverse changes in gut microbiota structure and obvious reductions in taxonomic richness and diversity [9]. This disruption of the intestinal barrier subsequently enhanced the permeability of the epithelial cell layers. The compromised intestinal barrier allows toxins or bacteria to enter the systemic circulation, resulting in inflammation and infection [10]. The imbalance of gut microbiome is associated with a variety of adverse conditions, including inflammatory bowel disease (IBD), antibiotic-associated diarrhea (AAD), *Clostridioides difficile* infection (CDI), obesity and other diseases, contributing to poor quality of life and high medical costs [11,12,13,14]. Therefore, it is urgent to restore intestinal homeostasis as soon as possible.

To alleviate the side effects associated with antibiotic treatment, effective interventions are necessary. Utilizing appropriate probiotics to regulate gut dysbiosis and related disorders represents a key strategy for addressing these challenges. Previous reports have shown that *Bifidobacterium* species have the function of relieving AAD, restoring gut microbiota homeostasis, and promoting the production of SCFAs [15,16]. Furthermore, *B. lactis* NFBAL23 markedly alleviated LPS-induced inflammation in neonatal rats [17]. We have obtained a bacterium named *Bifidobacterium animalis* subsp. *lactis* PB200 (*B. lactis* PB200), which has a good high-density culture characteristic and inhibitory effect on pathogenic bacteria in vitro. Notably, *B. lactis* PB200 has been reported to enhance immunity and improve cognitive flexibility [18,19]. However, its potential to regulate gut microbiota composition and alleviate intestinal damage following antibiotic-induced injury remains insufficiently explored. Therefore, the aim of this study is to systematically evaluate the effects of *B. lactis* PB200 on systemic inflammation, intestinal barrier integrity, gut microbiota diversity and composition, as well as bacterial-derived SCFA production in a murine model of antibiotic-induced intestinal injury. Moreover, correlation analyses between gut microbiota, inflammatory factors, and SCFAs levels were performed to further elucidate the underlying mechanisms. Understanding these effects of *B. lactis* PB200 may provide important insights into the development of probiotic-based therapeutic strategies for mitigating antibiotic-associated intestinal disorders in humans.

## 2. Materials and Methods

### 2.1. Materials

Enzyme-linked immunosorbent assay (ELISA) kits for LPS, IL-1β, IL-6, TNF-α and IL-10 were purchased from Meimian Industrial Co., Ltd. (Yancheng, China). de Man, Rogosa, and Sharpe (MRS) broth, MRS agar and L-cysteine hydrochloride was purchased from Hope Bio-technology Co., Ltd. (Qingdao, China). A BCA protein quantification kit was obtained from Solarbio (Beijing, China). Primary antibodies against ZO-1 (Cat.: 61-7300), Occludin (Cat.: 40-4700), Claudin-4 (Cat.: 36-4800) were purchased from Thermo Fisher Scientific Inc. (Waltham, MA, USA). Primary antibody against β-actin (Cat.: bsm-63325R), was obtained from Bioss Inc. (Beijing, China). Secondary antibodies, RIPA lysis buffer, protease and phosphatase inhibitor were purchased from Beyotime Biotechnology (Shanghai, China). Polyvinylidene fluoride (PVDF) membrane was purchased from Millipore Corp., (Bedford, MA, USA).

### 2.2. Bacteria Culture

The bacterial strain *Bifidobacterium animalis* subsp. *lactis* PB200 (CGMCC No.30390. China General Microbiological Culture Collection Center, Beijing, China) was cultured anaerobically in MRS broth supplemented with 0.05% (*w*/*v*) L-cysteine hydrochloride at 37 °C for 48 h. Prior to use, *B. lactis* PB200 was sub-cultured twice. The bacterial cells were then harvested by centrifugation at 4000× *g* for 10 min at 4 °C [20], and washed twice with sterile saline. In addition, in the pre-experiment to assess the approximate concentrations of viable bacteria, suitable dilutions of the culture were plated on MRS agar containing 0.05% (*w*/*v*) L-cysteine hydrochloride and incubated anaerobically at 37 °C for 48 h. Subsequently, the bacterial strain was diluted in sterile saline to prepare bacterial suspensions with viable count of 5 × 10^10^, 5 × 10^8^ and 5 × 10^6^ CFU/mL, respectively.

### 2.3. Animals and Experimental Design

Five-week-old male BALB/c mice (18 ± 2 g, SPF grade) were purchased from Beijing Vital River Laboratory Animal Technology Co., Ltd. (Beijing, China). All animals were maintained in a specific-pathogen-free environment (22 ± 2 °C, constant humidity 55 ± 5%, 12 h light/12 h dark cycle). All animals were housed in transparent Plexiglas cages (37 cm × 16 cm × 13.5 cm) and were allowed to receive food ad libitum (Research Diets, New Brunswick, NJ, USA) and water, and were acclimated for 1 week before the experiment.

**Figure 1:**
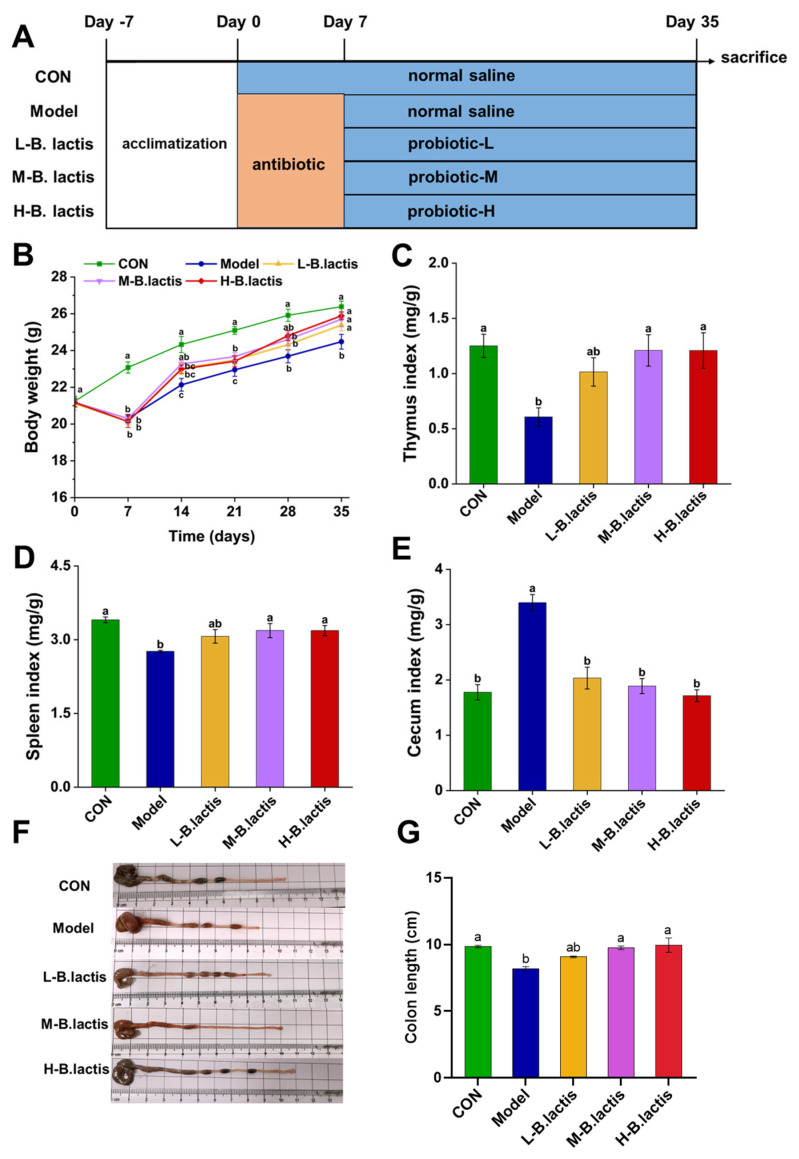
Effects of *B. lactis* on body weight and immune organ indices in mice. (**A**) Outline of study design. (**B**) Body weight of the mice. (**C**–**E**) Effects of *B. lactis* on spleen indices, thymus indices, and cecal indices in mice. (**E**–**G**) Effects of *B. lactis* on morphology of the cecum and colon length in mice. The results were expressed as the mean ± SEM, *n* = 6. Values with different lowercase letters are significantly different (*p* < 0.05).

Mice were randomly divided into 5 groups, with 10 mice in each group: control group (CON), treatment ceftriaxone sodium group (Model), treatment ceftriaxone sodium group + low-concentration bacterial suspension (L-*B. lactis*), treatment ceftriaxone sodium group + medium-concentration bacterial suspension (M-*B. lactis*), treatment ceftriaxone sodium group + high concentration bacterial suspension (H-*B. lactis*). A total of 50 mice were involved. The outline of the study design is shown in Figure 1A. CON received daily gavage of normal saline from days 0 to 35. The Model group received ceftriaxone sodium (2.5 g/kg BW) from days 0 to 7 followed by gavage of normal saline from days 7 to 35 to observe natural restoration. L-*B. lactis* group, M-*B. lactis* group and H-*B. lactis* group received gastric gavage of ceftriaxone sodium (2.5 g/kg BW) for a continuous period of 7 days followed by 0.2 mL of the *B. lactis* suspensions (1 × 10^6^, 1 × 10^8^, and 1 × 10^10^ CFU per day) from days 7 to 35, respectively. To minimize potential confounders such as spatial and temporal biases, cage positions were rotated daily, and sample collections were performed in a balanced order. After overnight fasting, all mice were fully anesthetized with isoflurane and then euthanized via cervical dislocation, blood sample, tissues and fecal samples were collected for analysis.

Spleen, thymus and cecal indices were calculated using the following formulas (1), (2) and (3):

(1) $$\mathrm{Spleen}\;\mathrm{index}(\mathrm{mg}/g)=\frac{\mathrm{Spleen}\;\mathrm{weight}\;(\mathrm{mg})}{\mathrm{Body}\;\mathrm{weight}\;(g)}$$

(2) $$\mathrm{Thymus}\;\mathrm{index}(\mathrm{mg}/g)=\frac{\mathrm{Thymus}\;\mathrm{weight}\;(\mathrm{mg})}{\mathrm{Body}\;\mathrm{weight}\;(g)}$$

(3) $$\mathrm{Cecal}\;\mathrm{index}(\mathrm{mg}/g)=\frac{\mathrm{Cecal}\;\mathrm{weight}\;(\mathrm{mg})}{\mathrm{Body}\;\mathrm{weight}\;(g)}$$

The animal experiment was approved by the Animal Ethics Committee of China Agricultural University (the ethical review serial number is AW41213202-4-2).

### 2.4. Serum Cytokines Measurements

Blood samples were collected from orbital sinus under anesthesia followed by centrifugation (4 °C, 3500 rpm, 10 min) to obtain serum. The levels of biomarkers including lipopolysaccharide (LPS), interleukin-1β (IL-1β), interleukin-6 (IL-6), interleukin-10 (IL-10) and tumor necrosis factor-α (TNF-α) were measured by commercial ELISA kits according to the manufacturer’s instructions. Briefly, serum samples were diluted prior to determination. After a series of incubation and washing steps, a substrate solution was added that reacted with the enzyme conjugated to the antibody. Subsequently, the absorbance was detected at a specific wavelength of 450 nm using a Microplate Reader (Bio-Rad Laboratories, Kyoto, Japan) within 15 min after adding a stop solution.

### 2.5. Histological Analysis of Colon Tissue

Histopathological evaluation of colonic architecture was conducted by Hematoxylin and Eosin (H&E) staining, as previously described [21]. The distal colon tissues (10 mm) of mice were collected and fixed in 4% paraformaldehyde solution immediately. After dehydration, tissues were embedded in paraffin and cut into 4 μm thick sections. Subsequently, the tissue sections were stained with H&E. Images were captured under a microscope (Axio Vert.A1, Carl Zeiss Microscopy GmbH, Oberkochen, Germany), and at least 3 images per section were analyzed. According to the severity of tissue edema (0−4), crypt damage (0−4), epithelial cell exfoliation (0−4), and inflammatory cell infiltration (0−4), the histology score was determined [22].

### 2.6. Western Blot Analysis

Western blotting was utilized to quantify the level of tight junctions, specifically ZO-1, Claudin-4, and Occludin. Colon samples were homogenized with RIPA lysis buffer that contained protease and phosphatase inhibitor. Ten micrograms of each sample were separated by 10% SDS-PAGE and transferred to polyvinylidene difluoride (PVDF) membranes. After being blocked by 5% skimmed milk, membranes were incubated overnight at 4 °C with specific primary antibodies against ZO-1 (1:1000), Occludin (1:1000), Claudin-4 (1:1000) and β-actin (1:5000). Subsequently, the corresponding secondary antibodies (1:1000) were incubated for 60 min. Immunoreactive bands were visualized using the enhanced chemiluminescent reagents. Images were taken by Tanon imaging system (Tanon, Shanghai, China), and the intensities of bands were quantified with Image J software (National Institutes of Health, Bethesda, MD, USA).

### 2.7. Microbial DNA Extraction and 16S rRNA Gene Sequencing

Microbial genomic DNA from fecal samples was extracted utilizing the E.Z.N.A.^®^ DNA Kit (Omega Bio-Tek, Norcross, GA, USA) in accordance with a recommended protocol. The DNA concentrations were detected using NanoDrop 2000 UV–Vis spectrophotometer (Thermo Scientific, Wilmington, DE, USA) followed by an assessment of DNA extraction quality. Subsequently, genomic DNA was employed to amplify the V3 to V4 regions of 16S rRNA genes. Paired-end sequencing was conducted on the Illumina MiSeq platform (Illumina, San Diego, CA, USA).

### 2.8. Bioinformatics Analysis

Microbiota data were processed and analyzed by QIIME2 (version 2019.4). Briefly, raw sequence data were demultiplexed and quality-filtered by QIIME followed by denoising with DADA2. Subsequently, the Greengenes database (Release 13.8) was used to annotate taxonomic information. According to the distribution of ASV in different samples, Alpha diversity in each group was assessed. To evaluate the Beta-diversity, principal component analysis (PCA) in ASV was estimated by R software. Then, remarkably different ASVs at each taxonomic level (Phylum, Class, Order, Family, Genus) were carried out using MetagenomeSeq of R software. The correlations between gut microbiota and serum biochemical parameters and fecal SCFAs were obtained using Spearman’s correlation analysis and the Benjamini–Hochberg procedure was applied for multiple corrections. The microbial functionality profiles were predicted based on PICRUSt2 (Phylogenetic Investigation of Communities by Reconstruction of Unobserved States) to generate the Kyoto Encyclopedia of Genes and Genomes (KEGG) pathway.

### 2.9. Fecal Short-Chain Fatty Acids Analysis

Fecal SCFAs concentrations were quantified as previously reported [23]. Briefly, Fecal samples were homogenized in 500 μL ultrapure water, and then 200 μL supernatant was extracted with 100 μL 15% (*v*/*v*) phosphoric acid and 280 μL ether. Subsequently, the samples were subjected to centrifugation (12,000 rpm, 4 °C 10 min), the supernatant was collected and measured by a gas chromatography–mass spectrometer (Thermo Fisher Scientific, San Diego, CA, USA).

### 2.10. Statistical Analysis

The IBM SPSS software (version 26.0, Chicago, IL, USA). was used for statistical analysis. All data were expressed as mean ± standard error of mean (SEM). The statistical analysis was performed with one-way ANOVA followed by Tukey’s multiple comparison test (*p* < 0.05).

## 3. Results

### 3.1. Effects of B. lactis on Body Weight and Immune Organ Indices in Mice with Antibiotic-Induced Intestinal Injury

The mice’s body weight and their food consumption were observed throughout the experiment. The body weights and average daily food intake of the mice in the Model, L-*B. lactis*, M-*B. lactis*, and H-*B. lactis* groups were significantly decreased in the second week (*p* < 0.05). The Model group had a lower weight than CON at the end of experiment. However, probiotic treatment recovered the body weight and food intake of the mice to normal level, and there was no significant difference in the mice’s body weight among the CON and groups L-*B. lactis*, M-*B. lactis* and H-*B. lactis* (Figure 1B and Figure S1). The thymus indices and spleen indices were presented in Figure 1C,D. Compared to CON, the thymus and spleen indices of the Model group were reduced remarkably (*p* < 0.05), which were reversed by probiotic treatment (*p* < 0.05). As shown in Figure 1E–G, the Model group had a higher cecal index than that in CON, while the length of the colon in the Model group was significantly shorter compared to that in CON (*p* < 0.05). Nevertheless, the cecal indices in groups L-*B. lactis*, M-*B. lactis* and H-*B. lactis* were dramatically decreased compared with that in Model group, and the colon lengths of mice in M-*B. lactis* group and H-*B. lactis* group were similar to those in CON (*p* > 0.05).

### 3.2. B. lactis Suppressed Inflammatory Response in Antibiotic-Induced Intestinal Injury Mice

**Figure 2:**
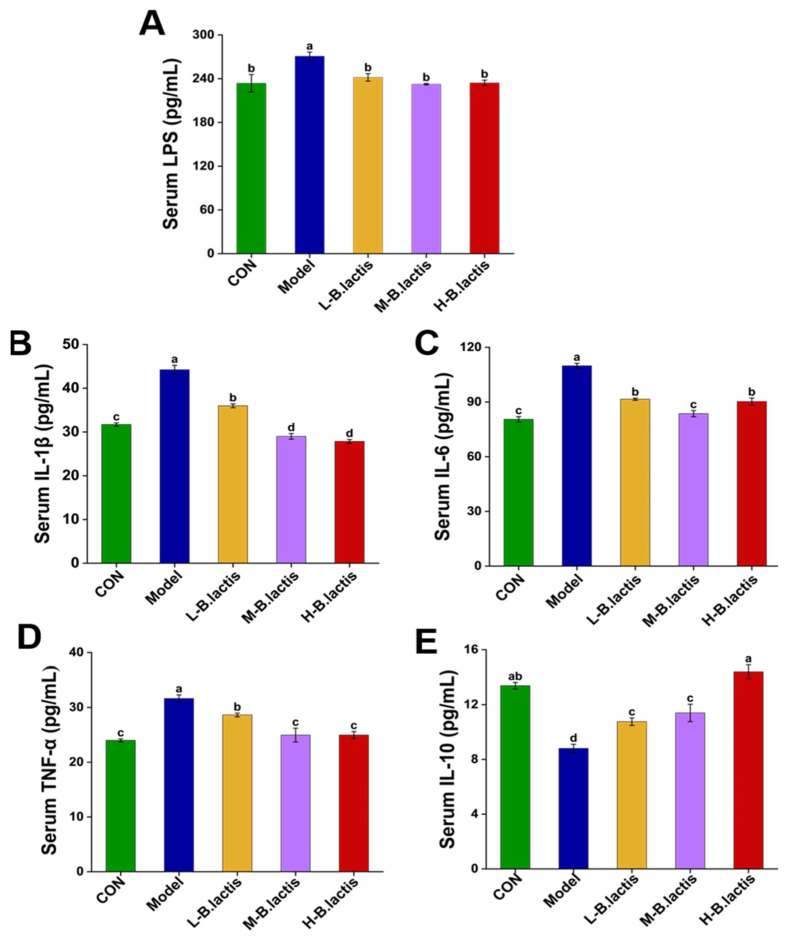
Effects of *B. lactis* on serum biomarkers in mice. (**A**) LPS, (**B**) IL-1β, (**C**) IL-6, (**D**) TNF-α, (**E**) IL-10. The results were expressed as mean ± SEM. *n* = 6. Values with different lowercase letters are significantly different (*p* < 0.05).

Compared to CON, mice in the Model group showed remarkably increased levels of different pro-inflammatory cytokines (*p* < 0.05), including IL-1β, IL-6 and TNF-α, along with LPS, and reduced IL-10, as shown in Figure 2. Notably, *B. lactis* PB200 treatment normalized these pro-inflammatory cytokines’ levels and mitigated the increase in LPS concentrations (*p* < 0.05). Furthermore, groups L-*B. lactis*, M-*B. lactis* and H-*B. lactis* had higher levels of IL-10 compared to the Model group (*p* < 0.05). *B. lactis* PB200 recovered serum pro-inflammatory and anti-inflammatory patterns challenged by an antibiotic.

### 3.3. Effects of B. lactis on Intestinal Barrier Integrity in Antibiotic-Induced Intestinal Injury Mice

**Figure 3:**
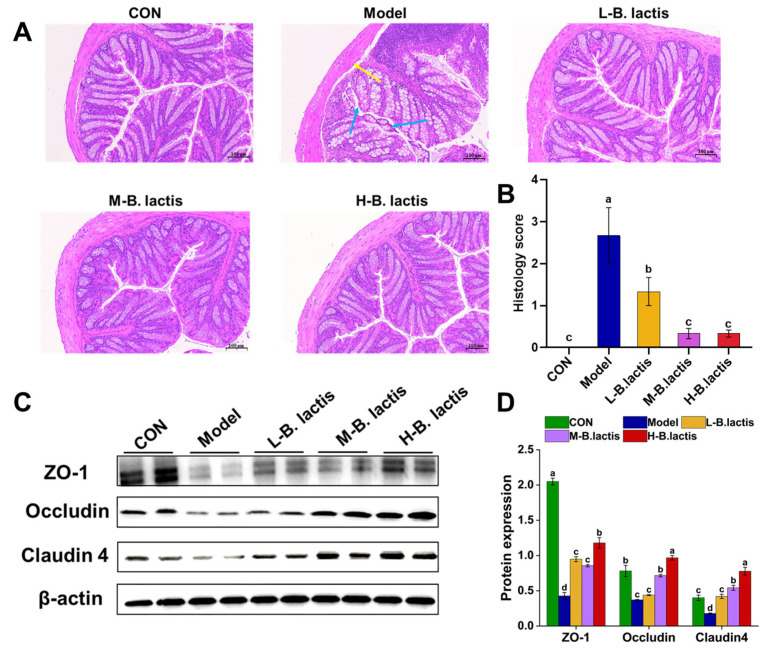
*B. lactis* enhanced the intestinal barrier function in mice. (**A**) Histopathological analysis with H&E staining was performed in the colon sections (*n* = 3). The images were taken at 20× magnification. Scale bars: 100 μm. The blue arrow showed the epithelial cells of the mucosa (necrotic and exfoliated), and the yellow arrow showed the gap between the mucosa and submucosa. (**B**) Histology score. (**C**,**D**) Protein expression of ZO-1, Occludin, Claudin-4. The results were expressed as mean ± SEM, *n* = 6, Values with different lowercase letters are significantly different (*p* < 0.05).

The colonic histological morphology of mice was evaluated by H&E staining. As shown in Figure 3A, neither antibiotic treatment nor probiotic supplementation significantly affected the crypt architecture of the colon. Furthermore, the examination of the mucosal structure revealed a notable absence of inflammatory cell infiltration within the lamina propria. The mice in the Model group showed shedding of their colonic epithelial cells and the mucosal boundaries were loose and irregular (Figure 3A; see bule arrows). The gap between the submucosa and muscular layer increased slightly (Figure 3A, see the yellow arrow), indicating that ceftriaxone sodium intervention might affect the intestinal mechanical barrier. However, compared with the Model group, supplementation of *B. lactis* PB200 effectively restored the integrity of the intestinal barrier, with significant reductions in the histology scores (*p* < 0.05 in all three probiotic groups, Figure 3B). We further investigated the effects of antibiotic treatment on the intestinal mechanical barrier, and the expression of ZO-1, Occludin and Claudin-4 was evaluated by Western Blot. As shown in Figure 3B,C, the expression of ZO-1, Occludin, and Claudin-4 of colon in Model group was down-regulated compared to that in CON (*p* < 0.05). Nevertheless, the expression of ZO-1 and Claudin-4 of colon tissues was markedly increased after probiotic supplementation (*p* < 0.05).

### 3.4. Effects of B. lactis on Fecal Short-Chain Fatty Acids (SCFAs) Levels in Antibiotic-Induced Intestinal Injury Mice

**Figure 4:**
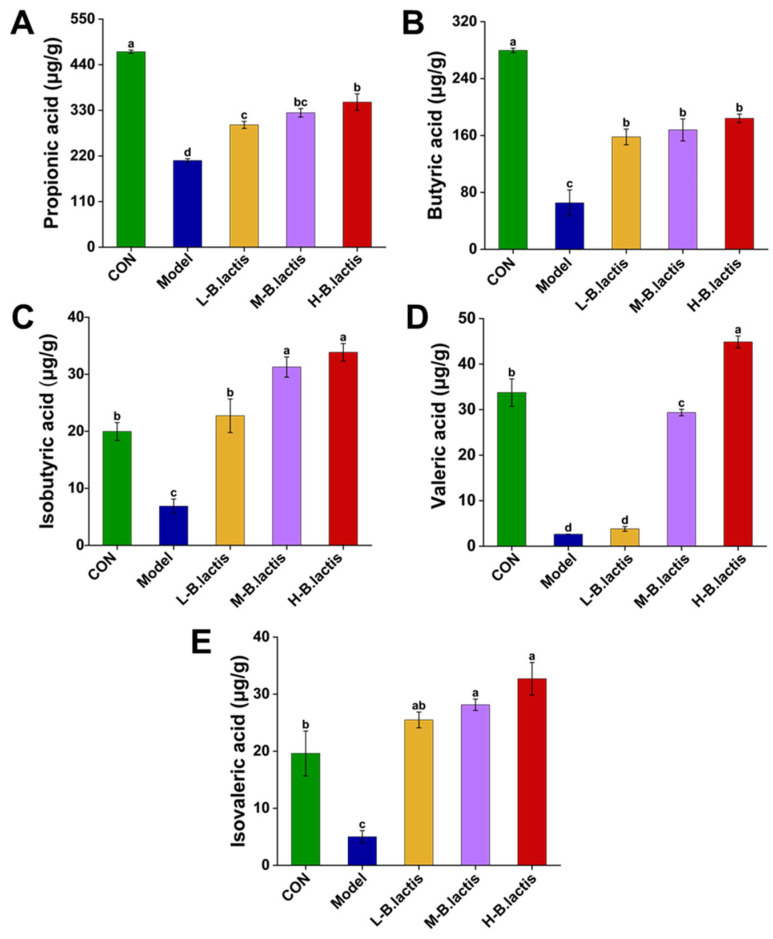
Effects of *B. lactis* on fecal SCFAs levels in mice. Concentrations of (**A**) propanoic acid, (**B**) butyric acid, (**C**) isobutyric acid, (**D**) valeric acid, and (**E**) isovaleric acid. The results were expressed as mean ± SEM, *n* = 6. Values with different lowercase letters are significantly different (*p* < 0.05).

As shown in Figure 4A–E, the propionic acid, butyric acid, isobutyric acid, valeric acid and isovaleric acid content in feces were significantly reduced in the Model group as compared to CON (*p* < 0.05). However, the supplementation of *B. lactis* PB200 reversed the reduction of these fecal SCFAs (*p* < 0.05).

### 3.5. Effects of B. lactis on Microbiota Diversity in Mice with Antibiotic-Induced Intestinal Injury

#### 3.5.1. Alpha Diversity

**Figure 5:**
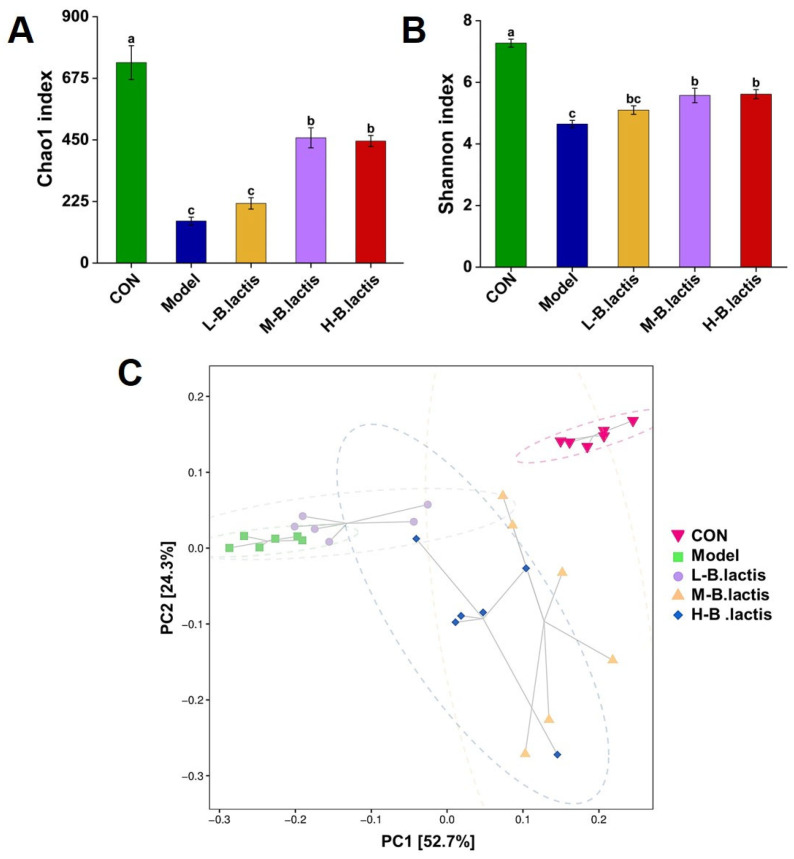
Effects of *B. lactis* on microbiota diversity in mice. Analysis of alpha diversity including (**A**) Chao 1 index and (**B**) Shannon index. (**C**) Beta diversity presented as principal component analysis (PCA). The results were expressed as mean ± SEM, *n* = 6. Values with different lowercase letters are significantly different (*p* < 0.05).

As shown in Figure 5A,B, the Chao1 index and the Shannon’s index in the Model group were significantly decreased compared to those in CON, suggesting that the microbial diversity was significantly reduced in the Model group (*p* < 0.05). While these indices were significantly increased after probiotic treatment (*p* < 0.05), the Chao1 index increased from 4.64 ± 0.12 to 5.62 ± 0.15 and the Shannon index increased from 153.41 ± 14.43 to 217.91 ± 20.72. These results suggested that probiotic treatment had a protective effect on microbial diversity.

#### 3.5.2. Beta Diversity

PCA analysis revealed that the microbial communities were distinctly separated among all groups, as well as the samples in the groups that successfully gathered in a certain range. PC1 percent variation explained 52.7% and PC2 percent variation explained 24.3%. The distance between CON and the Model group was greater, while the sample distribution of the M-*B. lactis* group was closer to that of CON (Figure 5C). These findings demonstrated the capacity of *B. lactis* PB200 to ameliorate antibiotic-induced dysbiosis through structural reconstitution of the gut microbiota.

### 3.6. B. lactis Modulated Gut Microbiota Composition in Mice with Antibiotic-Induced Intestinal Injury

**Figure 6:**
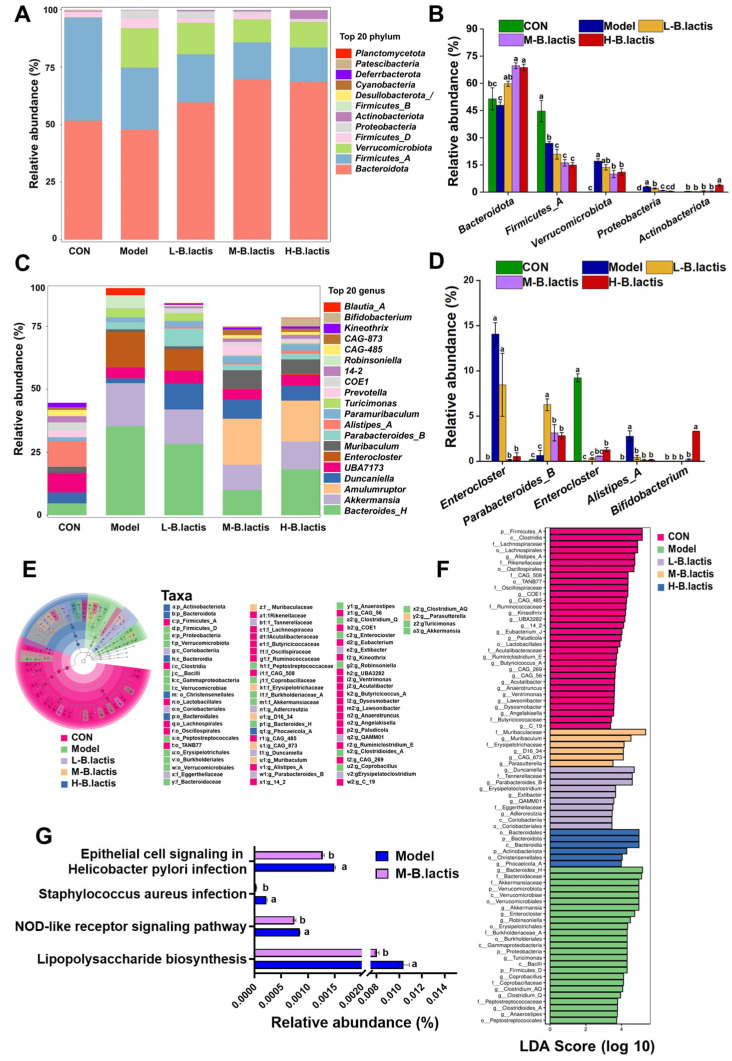
*B. lactis* modulated gut microbiota composition in mice. Overall taxonomic diversity of gut bacteria analyzed by LEfSe. (**A**) Composition of the gut microbiota at the Phylum level. (**B**) The relative abundance of *Bacteroides*, *Firmicutes*, *Verrucomicrobiota*, *Proteobacteria*, and *Actinobacteriota*. (**C**) Composition of the gut microbiota at the Genus level. (**D**) The relative abundance of *Enterocloster*, *Parabacteroides*, *Alistipes, Blautia* and *Bifidobacterium*. (**E**) Taxonomic cladogram plot from LEfSe analysis. (**F**) LDA score plot. An LDA score >2.0 was considered significant (*p* < 0.05). (**G**) Comparison of gut microbiota function prediction based on the KEGG pathway between the Model and M-*B. lactis* groups. The results were expressed as the mean ± SEM, *n* = 6. Values with different lowercase letters are significantly different (*p* < 0.05).

The ASV profile was estimated at different taxonomic resolutions and relevant differences among groups were revealed. At phylum level, in comparison to CON, the Model group exhibited a marked increase in *Proteobacteria* abundance (*p* < 0.05). However, the relative abundance of *Proteobacteria* was deceased while *Actinobacteorita* abundance was increased after probiotic intervention (*p* < 0.05). (Figure 6A,B). At the genus level, the Model group showed enrichment of *Enterocloster* and *Blautia_A* compared to CON. However, *B. lactis* PB200 supplementation significantly reduced the relative abundance of *Enterocloster* and *Blautia_A* (*p* < 0.05), and the relative abundance of *Parabacteroides_B* and *Alistipes_A* was significantly elevated (Figure 6C,D). Additionally, the relative abundance of *Bifidobacterium* was significantly enhanced in H-*B. lactis* groups. Linear discriminant analysis effect size (LEfSe) was also performed with the LDA score threshold of 2.0. As shown in Figure 6E,F, there were nineteen major significant different genera in the CON, mostly belonging to *Firmicutes_A*, *Lachnospiraceae*, *Rikenellaceae*, *Oscillospiraceae* and *Ruminococcaceae*. Ten major genera of *Proteobacteria*, *Firmicutes_D*, and *Bacteroidaceae* were enriched in the Model group. The L-*B. lactis* group had six significant distinct genera which mainly belonged to *Tannerellaceae*, *Eggerthellaceae* and *Coriobacteriia*. There were four notable different bacterial genera of *Muribaculaceae* and *Erysipelotrichaceae* in the M-*B. lactis* group. Moreover, *Bacteroidota* and *Actinobacteriota* were more abundant in the H-*B. lactis* group. KEGG metabolic pathway analysis in the microbial community was performed by PICRUSt2 analysis. As shown in Figure 6G, the KEGG metabolic pathway analysis emphasized remarkable alterations between the Model group and the M-*B. lactis* group. The microbial gene abundance in the epithelial cell signaling pathway in *Helicobacter pylori* infection, *Staphylococcus aureus* infection, Lipopolysaccharide biosynthesis, and NOD-like receptor signaling pathway was obviously increased in the Model group (*p* < 0.05). However, after *B. lactis* PB200 supplementation, the abundance of signaling pathways significantly decreased (*p* < 0.05).

### 3.7. Correlation Analysis of Gut Microbiota with Representative Inflammatory Cytokines and Short-Chain Fatty Acids

**Figure 7:**
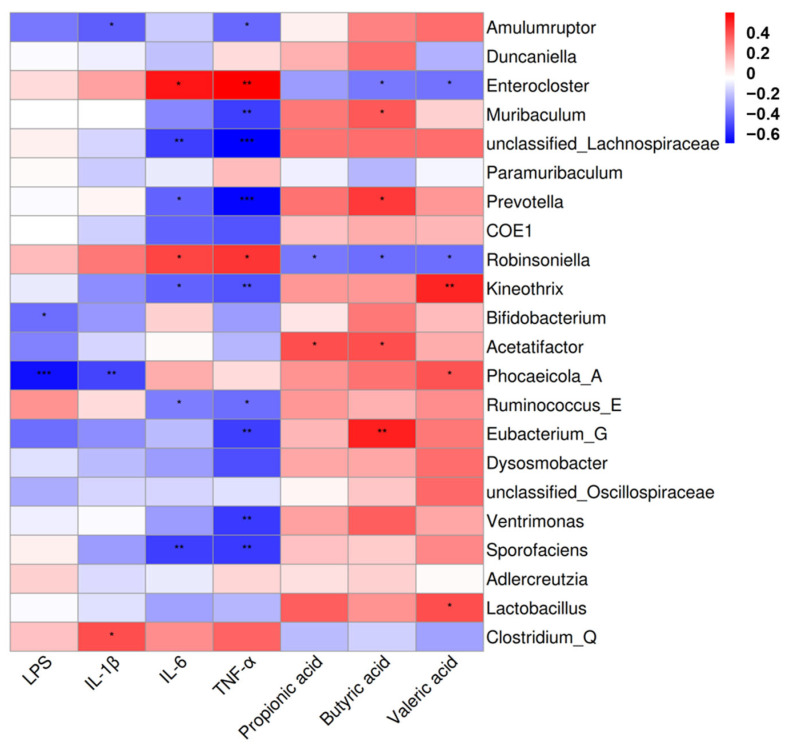
Heatmap of Spearman’s correlation of gut microbiota, representative inflammatory factors and short-chain fatty acids. Red represents a positive correlation, while blue represents a negative correlation. Significant correlations were noted by 0.01 < *p* ≤ 0.05 *, 0.001 < *p* ≤ 0.01 **, *p* ≤ 0.001 ***, following adjustment using the Benjamini–Hochberg procedure. *n* = 4.

The correlation analysis of 22 genera and inflammatory factors as well as SCFAs was performed based on Spearman’s correlation analysis. The results in Figure 7 showed that *Enterocloster* and *Robinsoniella* demonstrated positive correlations with serum IL-6 and TNF-α (*p* < 0.05). *Clostridium_Q* demonstrated positive correlations with serum IL-1β (*p* < 0.05). *Muribaculum*, *Prevotella*, and *Eubacterium_G* demonstrated positive correlations with fecal butyric acid (*p* < 0.05). *Acetatifactor* demonstrated positive correlations with fecal propanoic acid (*p* < 0.05). *Kineothrix*, *Phocaeicola_A* and *Lactobacillus* demonstrated positive correlations with fecal valeric acid (*p* < 0.05). In contrast, *Bifidobacterium*, and *Phocaeicola_A* showed negative associations with serum LPS (*p* < 0.05). *Prevotella, Ventrimonas* and *Sporofaciens* showed negative associations with serum TNF-α (*p* < 0.05). *Enterocloster* and *Robinsoniella* showed negative associations with fecal butyric acid and valeric acid (*p* < 0.05). These results indicated that *B. lactis* PB200 had an improved effect on antibiotic-induced intestinal injury in mice, which was related to the structural optimization of the gut microbiota.

## 4. Discussion

The widespread use of antibiotics not only significantly increases drug resistance but also disrupts gut microecology and induced intestinal damage [24,25]. Additionally, previous studies suggested that antibiotic treatment led to growth retardation and atrophy of immune organs in mice [26]. In the present study, we used ceftriaxone sodium to successfully establish an antibiotic-induced intestinal injury model in mice. We found that mice in the Model group were growth-retarded and had a lower thymus index and spleen index than CON (Figure 1A–C). The cecum of mice was notably enlarged in the Model group, with an increased cecal index (Figure 1D,E). However, all of these symptoms were restored after *B. lactis* PB200 supplementation, suggesting that it may mitigate the adverse effects of antibiotics on intestinal health. Our data also demonstrated the anti-inflammatory effects of *B. lactis* PB200 and its potential to restitute intestinal barrier function and modulate gut microbiota dysbiosis, suggesting its potential as a probiotic for gut health.

### 4.1. B. lactis PB200 Improved Intestinal Barrier Integrity and Gut Microbiota Dysbiosis in Antibiotic-Induced Intestinal Injury Mice

The mechanical barrier is mainly composed of an intestinal epithelial cell layer and tight junctions between cells [27]. After antibiotic treatment, the histological morphology of the intestine in mice changed significantly. For example, the submucosal layer of the intestines in mice exhibited swelling and edema, leading to a wider gap between the mucosa and the submucosa [28], suggesting that the intestinal mucosa was damaged. Similarly, our results showed that the intestinal epithelium of mice in Model group was damaged and the gap between submucosa and the muscular layer were increased (Figure 3A). Previous studies have shown that the intestinal epithelial barrier was impaired in patients after treatment with β-lactam antibiotics [29]. Antibiotics decreased the expression of tight junctions and increased intestinal paracellular permeability [30]. In this study, supplementation with *B. lactis* PB200 led to a significant increase in the expression of ZO-1, Occludin, and Claudin-4, compared to the Model group. These results indicated that *B. lactis* PB200 may contribute to maintaining gut integrity and improving intestinal barrier function. *Bacteroidota* could produce SCFAs and play a potential role in promoting host health [31]. *Proteobacteria*, a microbiological marker of gut microbiota dysbiosis, has been related to metabolic disorders, immune dysregulation, and intestinal inflammation [32]. Our results showed that the diversity and richness of the gut microbiota in Model group were remarkably reduced (Figure 5). Furthermore, the reduction in *Bacteroidota* alongside the abnormal enrichment of *Proteobacteria* in Model group mice collectively reflected the gut microbiota imbalance and disruption of intestinal homeostasis. Nevertheless, *B. lactis* PB200 supplementation increased the diversity and richness of the gut microbiota. *B. lactis* PB200 increased the relative abundance of *Bacteroidota* and restored the relative abundance of *Proteobacteria* to a normal level (Figure 6A,B), indicating that *B. lactis* PB200 supplementation alleviated gut microbiota dysbiosis. Furthermore, *Enterocloster* is a pathogen that has been linked to gut microbiota dysbiosis and human diseases such as IBD [33]. The colonization of *Enterocloster* in the intestines of mice was evident after antibiotic treatment [34]. *Blautia* was found to be significantly positively associated with IL-6, IL-1β, and TNF-α [35], and it was considered a potential biomarker of AAD [11]. Previous reports suggested that *Parabacteroides* reduced inflammation and strengthened intestinal barrier function, and was considered to be a “next-generation probiotic” [36]. Moreover, β-lactam antibiotics reduced the abundance of *Bifidobacterium* and *Parabacteroides* of term neonates [37]. In LPS-injury mice, the relative abundance of *Alistipes* was significantly reduced [38]. Furthermore, Grazul H et al. found that probiotics did not appear to colonize the intestine themselves. However, probiotic supplementation did significantly alter the types of bacteria in the gut of mice [39]. Consistent with these findings, we observed that the abundances of *Enterocloster* and *Blautia_A* were increased in the Model group (Figure 6E,F). Conversely, these changes were reversed with the supplementation of *B. lactis* PB200, along with a significant increase in *Parabacteroides_B, Bifidobacterium* and *Alistipes_A* (Figure 6C,D). However, relying solely on fecal sample analysis is insufficient for comprehensively evaluating the colonization of *Bifidobacterium lactis* PB200 within the intestinal tract. Future investigations could employ molecular techniques, such as fluorescent protein tagging, to observe the adhesion and distribution of *B. lactis* PB200 in the murine intestinal mucosa [40].

### 4.2. B. lactis PB200 May Restore Gut Homeostasis by Promoting SCFAs-Producing Bacteria and Enhancing Fecal SCFAs

Gut microbiota and its derived SCFAs played a crucial part in intestinal homeostasis. The disruption of the intestinal biological barrier indirectly impacted the function of the intestinal mechanical barrier, resulting in a decrease in the integrity of the intestinal barrier [41]. Existing evidence demonstrates that broad-spectrum antibiotics significantly impair intestinal barrier integrity by downregulating tight junctions (ZO-1 and Occludin) and reducing fecal SCFAs [42]. Studies using pseudo-germ-free mouse models and fecal microbiota transplantation (FMT) revealed that *Bifidobacterium breve* CCFM683 elevated the abundance of *Odoribacter splanchnicus*, a butyrate-producing bacterium. This microbial shift enhanced tight junctions expression, likely mediated by increased butyrate availability [43]. The addition of the probiotic caused a metabolic shift and the transcriptional response of the gut microbiota was modulated [44]. Notably, *Alistipes*, a recognized SCFAs producer, exhibited strong positive correlations with butyrate levels [45], highlighting its potential role in barrier maintenance. It has been reported that *Alistipes* is negatively linked to IBD and modulates the host lipidome to suppress colitis in an IL-10 deficient mouse model [46]. In line with these observations, our study identified significant positive correlations between fecal SCFAs content and the relative abundances of *Prevotella*, *Acetatifactor*, and *Lactobacillus*. Antibiotic treatment in Model group markedly reduced SCFAs concentrations. However, supplementation with *Bifidobacterium lactis* PB200 reversed this decline (Figure 4), potentially through its enrichment of SCFAs-producing taxa such as *Alistipes* and *Prevotella*. Studies on the gut health impacts of these specific bacteria are needed. These findings suggested that *B. lactis* PB200 ameliorated antibiotic-induced intestinal barrier dysfunction via restoring gut microbiota homeostasis by promoting SCFAs-producing bacteria and enhancing fecal SCFAs to improve tight junction integrity. While these data implicated microbiota-driven SCFAs production in PB200’s protective effects, further studies are required. Targeted metagenomic profiling and germ-free mouse models could clarify whether specific taxa directly mediate barrier repair through SCFAs synthesis.

### 4.3. B. lactis PB200 Had the Potential to Inhibit Inflammatory Pathways to Alleviate Intestinal Injury

Gut microbiota affects gut health by regulating the expression of inflammatory signaling pathways and key associated proteins. In our study, KEGG enrichment analysis indicated that the gene abundances in Lipopolysaccharide biosynthesis pathway and the NOD-like receptor signaling pathway were significantly different between the Model group and the M-B. lactis group. Lipopolysaccharide (LPS) has detrimental effects on the function of the intestinal barrier [47]. Toll-like receptors (TLRs) are widely expressed in the intestinal epithelium. LPS could activate TLR4, resulting in the generation of pro-inflammatory cytokines and chemokines that compromised the integrity of the intestinal epithelial barrier [48]. Broad-spectrum antibiotics exposure led to a remarkable increase in intestinal mucosal TLR4 reactivity [49]. However, probiotics such as *B. dentium* N8 significantly enhanced the mRNA expression levels of ZO-1 and Occludin, while concurrently downregulating the mRNA expression level of TLR4 [50]. In this study, we found that the content of serum LPS was significantly increased and the levels of serum inflammatory factors, namely IL-1β, IL-6 and TNF-α, were increased significantly after ceftriaxone sodium treatment (Figure 2). The NLRP3 inflammasome was activated in both the broad-spectrum antibiotic-treated Caco-2 cell model and mice, resulting in decreased expression of Claudin and morphological destruction of ZO-1 [51]. The reduction of SCFAs is indispensable for the activation of NLRP3 inflammasome and the production of IL-1β [52]. Gut microbiota dysbiosis resulted in excessive NF-κB activation, contributing to chronic inflammation. TLRs signaling activated the inflammatory NF-κB signaling pathway, upregulating NLRP3 and pro-IL-1β [53]. However, *B. lactis* PB200 supplementation modulated gut microbiota dysbiosis, decreased serum LPS levels, and increased levels of fecal SCFAs. We hypothesized that *B. lactis* PB200 transiently enhanced functions that could potentially promote anti-inflammatory pathways of the resident microbes. There were certain limitations in our research; further investigation is needed to determine whether *B. lactis* PB200 improved antibiotic-induced intestinal injury by blocking the activation of NLRP3 inflammasome as well as the NF-κB signaling pathway.

Taken together, the research results found that there was a dose-dependent effect of *B. lactis* PB200 supplementation on intestinal injury induced by an antibiotic. Notably, medium-dose *B. lactis* PB200 supplementation demonstrated superior efficacy in enhancing body weight and restoring gut microbiota diversity in mice, compared to the low dose. Although high-dose *B. lactis* PB200 had a more significant protective effect on the intestinal barrier, its impact on reducing inflammatory response and modulating gut microbiota was comparable to that of the medium dose. Therefore, medium-dose *B. lactis* PB200 could achieve a preferable overall improvement effect. Clinical studies are needed to confirm the beneficial effects of *B. lactis* PB200. Additionally, we did not explore the long-term effects of *B. lactis* PB200 supplementation, nor did we examine its potential to improve other intestinal diseases. Future research should address these aspects to fully evaluate the clinical potential of *B. lactis* PB200.

## 5. Conclusions

In conclusion, antibiotic treatment seriously disrupted the structure and composition of the gut microbiota, leading to systemic inflammation and impaired intestinal barrier integrity. *B. lactis* PB200 exhibited remarkable efficacy in ameliorating the gastrointestinal adverse effects induced by antibiotics and in restoring the balance of gut microbiota. Our data suggested that the amelioration of antibiotic-induced intestinal injury following *B. lactis* PB200 supplementation was collectively attributed to several mechanisms: the regulation of the inflammatory response, upregulation of tight junction expression, modulation of the gut microbiota, and enhancement of short-chain fatty acid production. The results support the idea that *B. lactis* PB200 could serve as a novel probiotic to alleviate antibiotic-induced intestinal injury. Future studies should explore the mechanistic basis using germ-free or knockout models. In addition, given that the physiological responses observed in animal experiments cannot be fully extrapolated to humans, the beneficial function of *B. lactis* PB200 on human health needs further clinical verification.

## Data Availability

The data that support the findings of this study are available from the corresponding author upon reasonable request.

## Supplementary Materials

The following supporting information can be downloaded at https://www.mdpi.com/article/10.3390/nu17101610/s1, Figure S1: The average daily food intake in each group.
