# Supplementary material for: Bifidobacterium animalis Subsp. lactis PB200 Improves Intestinal Barrier Function and Flora Disturbance in Mice with Antibiotic-Induced Intestinal Injury
Source: Nutrients. 2025 May 8;17(10):1610. doi: 10.3390/nu17101610 (PMC12114031; doi:10.3390/nu17101610)
Supplement: Supplementary file 1 [file nutrients-17-01610-s001.zip › nutrients-3568512-supplementary.pdf]

Supplementary Materials:

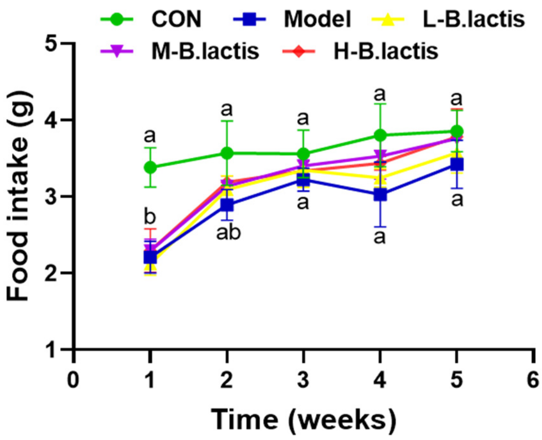

Figure S1. The average daily food intake in each group. The results were expressed as mean ± SEM, *n*=6. Values with different lowercase letters are significantly different ($p < 0.05$).
